# Lidocaine as a potential anti-seizure therapy for pediatric FIRES: a promising therapeutic approach

**DOI:** 10.3389/fped.2025.1696294

**Published:** 2025-12-17

**Authors:** Ying Yang, Ningning Wei, Qiao Guan, Han Xie, Xiaofang Huang, Xifang Ru, Ye Wu, Ying Wang, Tian Sang

**Affiliations:** Children’s Medical Center, Peking University First Hospital, Beijing, China

**Keywords:** lidocaine, pediatric, FIRES, status epilepticus, treatment

## Abstract

Febrile infection-related epilepsy syndrome (FIRES) is a catastrophic, drug-resistant epileptic encephalopathy characterized by acute onset following febrile illness and progression to (super-)refractory status epilepticus. We report a case of FIRES in a previously healthy 5-year-and-11-month-old boy who developed super-refractory status epilepticus (SRSE) on day 6 of a nonspecific febrile illness. The seizures were characterized by upward eye deviation, generalized tonic posturing, perioral cyanosis, and persistent impairment of consciousness. Despite comprehensive treatments including multiple immunotherapies (intravenous immunoglobulin administration, methylprednisolone pulse therapy, tocilizumab, and anakinra), ketogenic diet, multiple anti-seizure medications, and management of complications (intracranial pressure control, anti-infective therapy, and respiratory support), seizures remained refractory. Due to refractory seizures, intravenous lidocaine (3 mg/kg/h) was initiated after failed trials of midazolam, propofol and chloral hydrate, and only partial response to ketamine. With a consecutive 35-day infusion (cumulative duration >45 days), this regimen produced substantial reductions in both seizure frequency and severity, with clinically significant improvement in seizure control. Electroencephalogram (EEG) monitoring showed a decrease in the frequency and intensity of epileptic discharges, and improved background activity. The patient's level of consciousness also showed signs of improvement. This case is notable as few reports exist on the use of lidocaine in FIRES patients. It highlights the potential of lidocaine as a valuable anti-seizure option in FIRES management, suggesting the need for further investigation into its safety, efficacy, optimal dosage, and treatment duration in a larger cohort of FIRES patients.

## Introduction

Febrile infection-related epilepsy syndrome (FIRES) represents a rare yet catastrophic neurological disorder characterized by acute-onset super-refractory status epilepticus (SRSE) emerging in temporal association with antecedent febrile illness in previously neurotypical pediatric populations (1). This mysterious epileptogenic condition has an estimated annual incidence of 1 in 100,000 pediatric cases. The prognosis of FIRES is extremely poor (2). Longitudinal studies have shown that the mortality rate during the acute phase exceeds 20%. Among survivors, nearly all develop drug-resistant epilepsy and suffer from severe neurocognitive impairments.

Current pathophysiological understanding remains incomplete, though multimodal investigations propose a multifactorial etiological framework. Putative mechanisms include molecular mimicry triggered by neurotropic viral pathogens (particularly human herpesvirus 6 and enteroviruses), dysregulated immune-mediated pathways evidenced by elevated cerebrospinal fluid (CSF) interleukin-6 and other pro-inflammatory mediators, along with genetic susceptibility through polymorphisms in innate immunity regulators (e.g., *SCN1A*, *POLG, PCDH19*) (3). Notably, the absence of consistent infectious or autoimmune biomarkers in serological/CSF analyses suggests potential novel pathogenic mechanisms requiring further elucidation through advanced neuroimmunological and metabolomic profiling. Emerging evidence from multicenter cohort studies reveals critical limitations in current therapeutic strategies (4): (1) absence of randomized controlled trials validating immunomodulatory efficacy; (2) pharmacokinetic challenges in cytochrome P450-inducing antiepileptic regimens during systemic inflammation; and (3) paradoxical aggravation of epileptogenesis through NMDA receptor-mediated excitotoxicity from prolonged anesthetic use. This therapeutic impasse has spurred investigation into precision medicine approaches, including real-time CSF cytokine-guided therapy (targeting IL-6/IL-17A), ketogenic diet initiation within 72 h of seizure onset, and experimental neuromodulation techniques (responsive neurostimulation, vagus nerve stimulation). The management of FIRES presents a formidable challenge with a restricted repertoire of treatment options (1, 2, 5–7). Consequently, there is an urgent necessity to explore novel treatment modalities for FIRES.

Lidocaine, a well-established local anesthetic, has been utilized in some epilepsy cases, particularly in the management of acute seizures (8). It exerts its antiepileptic effect by blocking voltage-gated sodium channels (9), thereby reducing neuronal excitability and suppressing epileptic discharges. As an anti-arrhythmic drug, lidocaine has been confirmed to exhibit sodium channel blocking effects since the 1950s and has now become a second-line medication for status epilepticus. Its mechanisms of antiepileptic action have been well elucidated, primarily by selectively blocking neuronal voltage-gated sodium channels, inhibiting sodium ion influx, and thus effectively reducing abnormal neuronal discharges. In complex pathological processes such as FIRES involving innate immune activation, neuroinflammation, and ion channel dysfunction, lidocaine demonstrates potential therapeutic value due to its unique pharmacological effects.

Although lidocaine has shown therapeutic potential in specific epilepsy contexts, its utilization in the management of FIRES remains largely unexplored, with limited case reports available in the literature. In this report, we present a clinical case of a FIRES patient who experienced a substantial and rapid improvement after receiving lidocaine infusion therapy. This observation not only adds to the sparse body of evidence regarding lidocaine's role in FIRES treatment but also underscores its potential as an innovative and effective therapeutic strategy for this challenging neurological disorder.

## Case presentation

### Patient information

A previously neurotypical 5-year-and-11-month-old boy presented with a 24-day history of acute progressive illness characterized by recurrent febrile seizures with convulsions and persistent altered consciousness. His developmental milestones were age-appropriate before disease onset, with no personal or family history of epilepsy or metabolic disorders.

### Clinical course before lidocaine use

The patient had a history of fever, with seizures commencing on the 6th day of fever. The initial seizures were manifested as upward deviation of both eyes, limb stiffness, lip cyanosis, unresponsiveness, and persistent impaired consciousness. Over the course of the illness, he continued to experience frequent seizures, which evolved to predominantly facial twitching with occasional limb movements.

Despite receiving a comprehensive spectrum of treatments, including multiple rounds of immunotherapy (such as methylprednisolone pulse therapy, tocilizumab treatment, anakinra treatment and multiple administrations of gamma globulin), a variety of anti-seizure medications (including levetiracetam, lacosamide, clobazam, perampanel, topiramate, etc.), sedative drugs (midazolam, propofol and chloral hydrate, and only partial response to ketamine) and treatments for complications such as intracranial pressure reduction, anti-infection, and respiratory management, the seizures persisted. Electroencephalogram (EEG) monitoring continuously detected electrical seizures or electroclinical status epilepticus, with a high seizure burden, often reaching 50%–100% during some monitoring intervals.

The patient also developed multiple complications during the disease course. Pneumonia was evident based on abnormal lung auscultation and chest x-ray findings of increased lung markings and bilateral pulmonary inflammatory exudation. Respiratory failure occurred, initially necessitating mechanical ventilation and later transitioning to high-flow nasal cannula oxygenation. Hematuria was present and managed with hydration and urine alkalization. Abnormal liver function, indicated by elevated liver enzymes, was treated with hepatoprotective agents. Urinary calculi were detected, and mild anemia, hyperbilirubinemia, electrolyte disorders (including low phosphorus, low magnesium, and low sodium), and hypoproteinemia were also present, all of which were managed symptomatically.

### Treatment with lidocaine

Due to the persistent and refractory nature of the seizures, lidocaine was introduced as an antiepileptic alternative. The initial dosage of lidocaine was meticulously titrated according to the patient's age, weight, and initial response. The patient received lidocaine infusion at a starting rate of 3 mg/kg/h, and the dosage was adjusted in accordance with seizure control and serum lidocaine levels.

**Figure 1:**
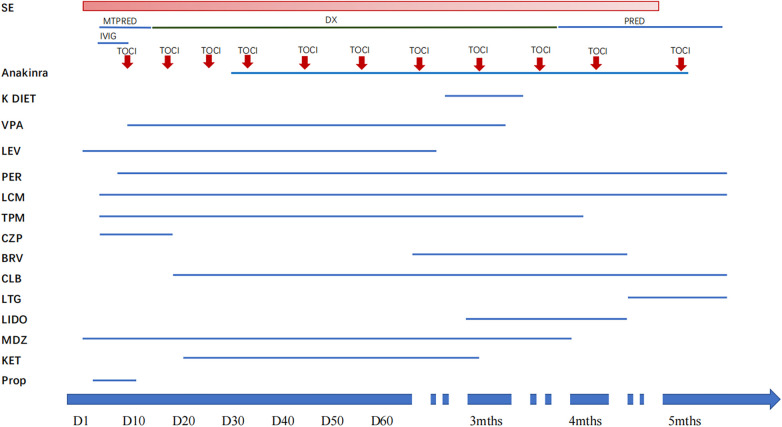
Pharmacological treatment profile of this patient: dosing and duration. BRV, brivaracetam; CLB, clobazam; CZP, clonazepam; DX, dexamethasone; IVIG, intravenous immunoglobulin; K DIET, ketogenic diet; KET, ketamine; LEV, levetiracetam; LCM, lacosamide; LTG, lamotrigine; LIDO, lidocaine; MDZ, midazolam; MTPRED, methylprednisolone; PER, perampanel; Prop, propofol; SE, status epilepticus; TPM, topiramate; TOCI, tocilizumab; VPA, valproate.

During the course of treatment (Figure 1), the patient received intravenous infusions of midazolam (maximum 20 μg/kg/min), propofol (maximum 5 mg/kg/h) and ketamine (maximum 5 mg/kg/h). While on lidocaine infusion, concurrent administration of midazolam (with a maximum combined dose of 3 ug/kg/min) and ketamine was maintained (with a maximum combined dose of 2.5 mg/kg/h). After combined use with lidocaine, the infusion rates of ketamine and midazolam decreased significantly compared with those before.

Over the 35-day period of lidocaine use, a significant improvement in seizure control was observed. The frequency of seizures decreased substantially. Prior to lidocaine treatment, the patient experienced multiple seizures per day, sometimes as frequently as every few hours. After the initiation of lidocaine, the seizure frequency decreased to only occasional seizures, sometimes only one or two seizures over a few days. The severity of seizures also diminished. The facial twitching became less intense, and the associated limb movements, which were more pronounced during seizures previously, became minimal.

**Figure 2:**
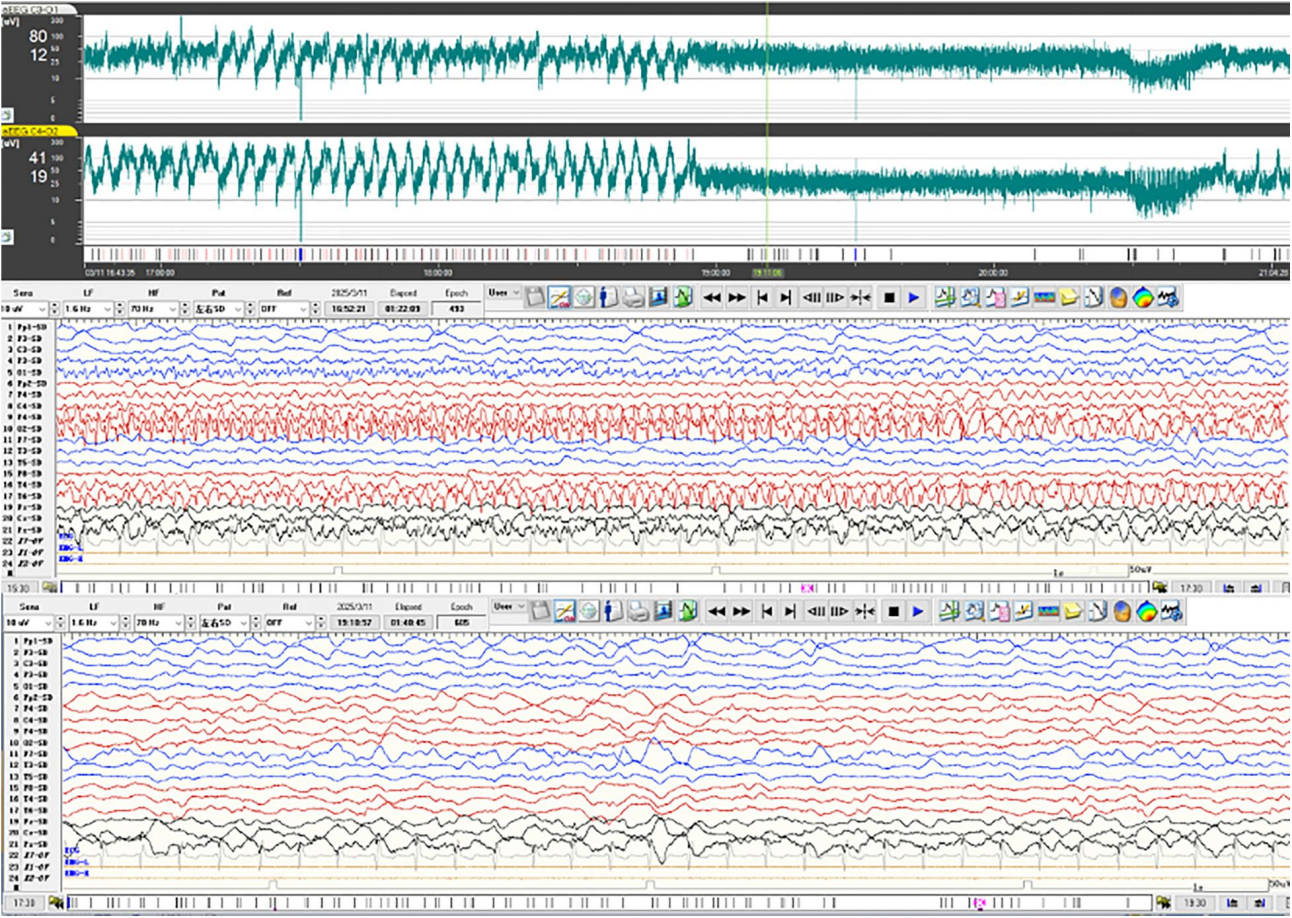
The video-electroencephalogram (VEEG) findings at 6 years and 3 months of age (3rd disease month) demonstrated predominant generalized delta slow wave activity with multifocal spike waves, sharp-and-slow wave complexes, and biphasic/positive sharp waves predominantly localized in frontal and temporal regions, accompanied by recorded electroclinical seizures with multifocal onset or electrographic status epilepticus, which ceased 2.5 h after lidocaine dose escalation. For the EEG with bilateral bipolar leads, the amplitude-integrated electroencephalography(aEEG) is displayed at a scale of 4.5 h per screen, and the VEEG is presented at 15 s per screen.

The patient's overall condition gradually stabilized. The EEG demonstrated a decrease in the frequency and intensity of epileptic discharges (Figure 2). The background activity on the EEG also improved, with a reduction in the amount of abnormal slow-wave activity that was prominent during seizure-free intervals. In addition to the improvement in seizure control, the patient's level of consciousness also showed signs of improvement. He became more responsive to stimuli, and the periods of unresponsiveness during non-seizure times decreased.

### Monitoring and follow-up

During the lidocaine treatment, the patient was closely monitored. Continuous electrocardiographic monitoring, including heart rate, blood pressure, and respiratory rate, were measured hourly initially and then at longer intervals as the patient's condition stabilized. Additionally, the patient's neurological status, including seizure frequency, seizure characteristics, and level of consciousness, was continuously evaluated by the medical team.

The EEG was repeated at regular intervals, typically every few days, to assess the response to lidocaine treatment. After 35 days of lidocaine treatment, the patient was gradually weaned off lidocaine. The weaning process was slow and carefully monitored to prevent seizure recurrence. During the weaning period, other anti-seizure medications were adjusted according to the patient's seizure control status.

Regular follow-up appointments were scheduled to monitor the long-term effects of lidocaine treatment and the recurrence of seizures. At follow-up visits, the patient's neurological status was re-evaluated, and additional tests, such as EEG and blood tests to assess liver and kidney function (since lidocaine is metabolized by the liver and excreted by the kidneys), were performed. The patient's development and quality of life were also evaluated, and appropriate support and rehabilitation measures were provided as needed.

The patient began to exhibit gradual recovery of consciousness at the age of 6 years and 5 months (the 5th month of disease course, and the 2nd month of lidocaine treatment), with seizure episodes gradually being controlled. At the last follow-up at 6 years and 6 months of age, the child had regained sound and visual-tracking abilities, could sit independently, but still showed no intentional vocalization.

## Discussion

This case report demonstrates the efficacy of lidocaine infusion in controlling seizures in a patient with FIRES. In the existing literature, most treatment strategies for FIRES focus on immunomodulation, ketogenic diet and traditional anti-seizure medications (4, 10–12). The use of lidocaine in this patient is particularly noteworthy as, to the best of our knowledge, there have been extremely few reports of its use in FIRES patients (13).

The mechanism by which lidocaine exerts its antiepileptic effect is well-understood at the cellular level (8). In the context of FIRES, where the underlying pathophysiology likely involves a complex interplay of immune activation, neuronal hyperexcitability, and inflammation, lidocaine's ability to directly target neuronal excitability offers a potentially valuable therapeutic approach. One of the key advantages of lidocaine in this case was its relatively rapid onset of action. In the early stages of lidocaine infusion, within a few hours to a day, a noticeable reduction in seizure frequency and severity was observed. This contrasts with some of the other immunomodulatory treatments used in FIRES, which often take days to weeks to show a significant effect. The rapid response to lidocaine allowed for a more immediate improvement in the patient's condition, alleviating the burden of continuous seizures on the brain and potentially minimizing long-term neurological damage.

Another advantage of lidocaine is its relatively favorable safety profile. Although lidocaine can have potential side effects, such as central nervous system depression, cardiovascular effects (14) (including hypotension and arrhythmias), and local tissue reactions, in this patient, these side effects were minimal. The close monitoring of serum lidocaine levels and vital signs during the treatment period ensured that the dosage was optimized and any potential side effects were promptly detected and managed (15). The patient did not experience any significant adverse events related to lidocaine treatment, further supporting its use in this challenging patient population.​ The adverse effects of intravenous lidocaine primarily involve the neurological and cardiovascular systems, demonstrating clear dose-dependency and infusion rate correlation (16). Neurotoxicity represents the most frequently observed category of adverse reactions, with tremor serving as a characteristic early clinical indicator. Additional manifestations include drowsiness, dizziness, dysarthria, and ataxia, which are typically reversible upon dose reduction or drug discontinuation. However, at elevated plasma concentrations, lidocaine may induce generalized seizures. Although less common, cardiovascular toxicity can manifest as severe complications including sinus bradycardia, sinus arrest, hypotension, and shock, particularly in cases of overdose or rapid administration.

However, it is important to note that this is a single case report, and the findings cannot be generalized to all FIRES patients. There is a need for further studies, particularly large-scale, well-controlled clinical trials, to confirm the safety and efficacy of lidocaine in a larger number of FIRES patients. Such trials should also aim to determine the optimal dosage, treatment duration, and potential predictors of response to lidocaine in FIRES. Additionally, future research could explore the combination of lidocaine with other existing treatments for FIRES, to determine if there are synergistic effects that could further enhance patient outcomes. The positive response to lidocaine in this patient suggests that it may be a valuable addition to the therapeutic arsenal for FIRES. Given the limited treatment options and poor prognosis associated with FIRES, the use of lidocaine could offer a new avenue for improving outcomes in these patients. Furthermore, the relatively favorable safety profile of lidocaine, when administered under careful monitoring, makes it a viable option for long-term management of refractory seizures in FIRES.

In the management of pediatric FIRES, intravenous lidocaine has demonstrated clinically meaningful utility as an adjunctive anticonvulsant and sedative agent. The pharmacological rationale for its application derives from its potent voltage-gated sodium channel blocking properties, which may interrupt the pathological hyperexcitability characteristic of FIRES.

## Conclusion

In conclusion, this case report demonstrates the therapeutic potential of lidocaine in the treatment of FIRES. The successful use of lidocaine in this patient suggests its promise as a treatment option for FIRES patients. Given the limited treatment options for FIRES and the high morbidity and mortality associated with this condition, these findings provide important insights into FIRES management. However, more research is required to fully understand the role of lidocaine in FIRES and to optimize its clinical use. Future studies should focus on validating these findings in larger patient populations, exploring the underlying mechanisms of action, and determining optimal strategies for integrating lidocaine into the existing treatment approaches for FIRES.

## Data Availability

The raw data supporting the conclusions of this article will be made available by the authors, without undue reservation.
